# Rac1: A Regulator of Cell Migration and a Potential Target for Cancer Therapy

**DOI:** 10.3390/molecules28072976

**Published:** 2023-03-27

**Authors:** Ning Ma, Erqian Xu, Qing Luo, Guanbin Song

**Affiliations:** 1 College of Bioengineering, Chongqing University, Chongqing 400030, China; 2 Key Laboratory of Biorheological Science & Technology, Ministry of Education, Chongqing University, Chongqing 400030, China

**Keywords:** Rac1, cell migration, invasion, metastasis, actin cytoskeleton, cancer therapy

## Abstract

Cell migration is crucial for physiological and pathological processes such as morphogenesis, wound repair, immune response and cancer invasion/metastasis. There are many factors affecting cell migration, and the regulatory mechanisms are complex. Rac1 is a GTP-binding protein with small molecular weight belonging to the Rac subfamily of the Rho GTPase family. As a key molecule in regulating cell migration, Rac1 participates in signal transduction from the external cell to the actin cytoskeleton and promotes the establishment of cell polarity which plays an important role in cancer cell invasion/metastasis. In this review, we firstly introduce the molecular structure and activity regulation of Rac1, and then summarize the role of Rac1 in cancer invasion/metastasis and other physiological processes. We also discuss the regulatory mechanisms of Rac1 in cell migration and highlight it as a potential target in cancer therapy. Finally, the current state as well as the future challenges in this area are considered. Understanding the role and the regulatory mechanism of Rac1 in cell migration can provide fundamental insights into Rac1-related cancer progression and further help us to develop novel intervention strategies for cancer therapy in clinic.

## 1. Introduction

Cell migration, a multiple-step, complex process influenced by various factors, is critical in body development, damage repair, immune response, vascular reconstruction, tumor invasion and metastasis, etc. Based on the cells’ living environment, cell migration can be divided into two-dimensional (2D) and three-dimensional (3D) migration. It can also be divided into single-cell and collective cell migration, according to the relationship between cells. According to cell morphology during migration, cell migration can be divided into mesenchymal and amoeboid modes of migration [1,2,3].There is no absolute limit between each migration mode, despite different cell types having preferred modes; cells always need to frequently change migration modes during migration, which makes the regulation of cell migration complicated [3]. The steps of cell migration mainly include: (1) the establishment of cell front–rear axis polarity; (2) the extension of cell migration leading edge; (3) formation of adhesion between cell and matrix; (4) contraction of cell uropod [2,4]. Cells migrate mainly in response to extracellular physical and chemical signals, which are primarily mediated by actin cytoskeleton [5]. Moreover, cells establish the front–rear axis polarity by a self-organized mechanism, which triggers cell migration [2]. This indicates cell migration can be driven by both internal and external signals to cells. Due to many influencing factors, the regulatory mechanisms of cell migration are very complex. It is critical to explore the regulatory mechanisms of cell migration so as to be able to intervene in multiple physiological or pathological processes of the body.

Ras-related C3 botulinum toxin substrate 1 (Rac1) is one of the central members of Rho GTPases. As a cytoskeleton regulatory protein, Rac1 regulates cell adhesion and movement mainly by promoting actin cytoskeleton remodeling, playing an important role in transferring migration signals to the cytoskeleton [6]. Rac1 widely exists in various cell types and is highly expressed in tumors. Increasingly, studies are reporting the role of Rac1 in tumor invasion or metastasis and are, therefore, suggesting that Rac1 is a potential target for tumor therapy [7]. In this review, we summarize the role of Rac1 in cell migration, tumor invasion and metastasis, and the regulatory mechanisms involved.

## 2. Molecular Structure and Activity Regulation of Rac1

**Figure 1:**
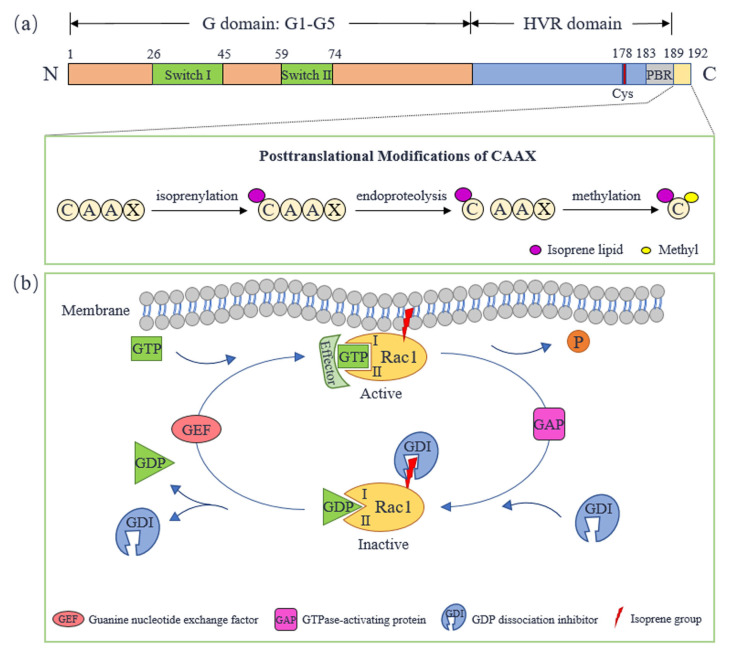
Molecular structure and activity regulation of Rac1. (**a**) The molecular structure of Rac1. (**b**) The cycle of Rac1 between active and inactive forms. Cys: cysteine; GEF: guanine nucleotide exchange-factor; GAP: GTPase-activating protein; GDI: GDP dissociation inhibitor; HVR: hypervariable region; PBR: polybasic region.

Rac1, initially identified in human platelets, is a kind of small GTP-binding protein and a member of the Rac subfamily of the Rho family in the Ras superfamily. Rac1 is named for its role as a substrate for botulinum C3 ADP ribosyltransferase [8,9]. The human Rac1 gene is located on chromosome 7p22, composed of 7 exons over a length of 29 kb [10]. The protein coded by the Rac1 gene comprises 192 amino acids, and its molecular weight is about 21 kDa. The N-terminal of Rac1 contains a conserved G-domain [11], consisting of five sequences, G1–G5 (Figure 1a), which is a basic functional unit of GTP-binding protein to bind or hydrolyze GTP [12]. Rac1 transforms between switch-ON state (GTP-bound form) and switch-OFF state (GDP-bound form). This is achieved through the conformational change in switch I and switch II, which involves amino acid residues 26 to 45 and 59 to 74, respectively [13,14]. The C-terminal of Rac1 contains a hypervariable region (HVR) [11] with a conserved cysteine residue at position 178, which is similar to Rac2 and Rac3. Downstream from the cysteine residue is a polybasic region (PBR) composed of polybasic amino acid residues. Rac1′s C-terminal sequence term is CAAX (C represents cysteine, A represents an aliphatic amino acid, and X represents any amino acid) [11,15,16], which is adjacent to PBR. CAAX is the site of Rac1 to undergo a series of posttranslational modifications. This is necessary for Rac1′s correct subcellular localization (mainly in plasma membrane), so that Rac1 can exert its biological function. The posttranslational modifications undergo three steps: (1) isoprenylation modification of cysteine residue in CAAX; (2) -AAX is cleaved from Rac1′s C-terminal which is catalyzed by endoprotease; (3) methylation modification of prenylated cysteine residue. These modifications enhance hydrophobicity to promote the localization of Rac1 on the plasma membrane [15].

As a GTP-binding protein, Rac1 transforms between an active GTP-bound state and an inactive GDP-bound state, performing as a molecular switch (Figure 1b). The process is regulated by guanine nucleotide-exchange factors (GEFs), GTPase-activating proteins (GAPs) and GDP dissociation inhibitors (GDIs). GEFs accelerate GDP/GTP exchange, which promotes Rac1 to activate and locate to the plasma membrane so that Rac1 interacts with downstream effectors. Rac1 can be activated by multiple GEFs including Tiam1, Vav2 and Trio. Both Tiam1 and Vav2 are expressed in a variety of insulin-secreting cells, promoting Rac1 to regulate islet function [17]. Tiam1 is a specific GEF of Rac1, which is highly expressed in the brain and can promote Rac1 to regulate neural function [18]. In addition, Tiam1-Rac1 signal transduction also plays a role in cancer initiation and progression [19]. Trio and Kalirin are unique GEFs because they contain two GEF domains, and are proved to regulate F-actin remodeling through activating Rac1, which plays a central role in neurodevelopment, excitatory synaptic transmission and tumor cell migration and invasion [20,21]. GAPs promote GTP hydrolysis, bringing Rac1 back to an inactive state. Sometimes, Rac1-GAP and Rac1-GEF act in concert to precisely regulate Rac1 signaling. Rac1-GAP Bcr can form a GEF/GAP complex with Tiam1 that is essential for controlling Rac1 activity during neuronal development [22,23]. GDIs interact with inactive Rac1 and hide its isoprene group to keep Rac1 inactive, which protects Rac1′s plasma membrane location [24,25]. The interaction between Rac1 and GEFs, GAPs or effectors are closely related to its switch I and switch II [26].

Endogenous Rac1 activity levels can be detected using an Rac1 fluorescence resonance energy transfer (FRET) biosensor [27]. Genetically encoded FRET-based sensors afford the view of spatiotemporal modular precision of Rac1-GTP [28]. Glutathione S-transferase (GST) or green fluorescent protein (GFP)-labeled PAK (p21 activated kinase)-RBD (Rac-binding domain) is an effective probe for detecting active Rac1. Its binding frequency with endogenous Rac1-GTP can be facilitated by rapid immunohistochemistry (R-IHC), so that Rac1 activation in cancer formalin-fixed paraffin-embedded (FFPE) tissues can be detected [29]. Rac1 activity can also be manipulated artificially. Wu et al. developed a new approach to produce photoactivatable Rac1 (PA-Rac1) reversibly and repeatedly, which generates precisely localized Rac1-GTP [30]. In addition to active forms, this optogenetic tool is also generated in a dominant negative (DN) form of Rac1, which is important for investigating Rac1 signaling during cell membrane protrusion and migration [31]. Moreover, optogenetic activation of Rac1 GEFs can activate Rac1 indirectly. Fusions of cryptochrome CRY2 with the DHPH catalytic domain of Rac1 GEFs such as Tiam1 can spatially and temporally recruit them to plasma membrane, thus producing a precisely spatial distribution of Rac1-GTP [32,33].

## 3. Rac1 Regulates Cell Migration and Tumor Invasion and Metastasis

### 3.1. Rac1 Regulates Invasion and Metastasis of Tumor Cells

Tumor cell invasion and migration is fundamental to tumor metastasis. The dynamic interaction between the cells and the extracellular matrix (ECM) is the key factor to drive tumor cell invasion and migration. In head and neck squamous cell carcinoma (HNSCC), Rac1 is involved in cell–matrix adhesion via fibronectin mediated by Ras-associated protein 1 (Rap1) [34]. The upregulation of Rac1 expression can promote the lamellipodia formation of hepatoma cells, facilitating hepatocellular carcinoma (HCC) metastasis [35], and this may correlate with the Scar/Wave complex, which is the main downstream target of Rac1. It has been reported that the Scar/Wave complex can drive lamellipodia formation and the migration of melanoma cells [36]. The Ras/Rac1/Wave2 signaling pathway also plays a role in osteosarcoma metastasis [37]. The cell adhesion and lamellipodia formation mediated by Rac1 involves the regulation of the cytoskeleton. Rac1 receives upstream signals and transfers them to the cytoskeleton, thus regulating tumor cell migration. Histone deacetylase 6 (HDAC6) is crucial in rhabdomyosarcoma progression. It promotes cell migration and invasion by regulating the cytoskeletal dynamics, in which Rac1 plays a critical role [38]. External mechanical stimulation regulates cytoskeletal remodeling and contraction through Rac1 and other cytoskeleton remodelers, thus maintaining pancreatic cancer cell movement [39]. In addition to promoting cytoskeletal polymerization in the cytoplasm to drive tumor cells to extend aggressive pseudopodia and enhance mobility, Rac1 can also play a role through nuclear translocation. One study has shown that abnormal nuclear accumulation of Rac1 leads to nuclear deformation and high RhoA activity in cytoplasm, which favors matrix degradation and the invasion of tumor cells [40].

Epithelial–mesenchymal transition (EMT) is the key mechanism of tumor metastasis. Denticleless E3 ubiquitin protein ligase homolog (DTL) promotes cervical cancer cell EMT through the Rac1-related signaling pathway [41]. Atypical protein kinase C-ι (aPKC-ι) is essential for colorectal cancer cell EMT. It mediates cell EMT and subsequent migration and invasion via Rac1/JNK activation [42]. A hypoxic environment in solid tumors is a powerful driver of tumor cell migration and invasion. Hypoxia can activate Rac1, accelerating gastric and breast cancer cell migration [43,44]. This may be relevant to EMT. One study has shown that after activation by hypoxia, Akt/Rac1 axis can upregulate Fascin-1 to promote EMT of liver cancer cells [45].

Increasing numbers of studies have shown that Rac1 is an important target in antitumor therapy. Some natural products with antitumor activities, such as zerumbone and genistein, inhibit tumor cell migration and invasion by suppressing Rac1 expression or the Rac1-related signaling pathway [46,47]. Lysine demethylase 6A (KDM6A) in bladder cancer cells can inhibit Rac1 activation to prevent cell mobility [48]. Pals1 is confirmed as a new regulator of the occurrence of cancer development. It functions as an Rac1 repressor to inhibit colorectal cancer cell migration [49].

These studies indicate that Rac1 plays critical roles in tumor cell invasion and metastasis, and the signaling pathways involved in these processes are complicated (as shown in Table 1). Clarifying the complex regulatory network and the role of Rac1 in tumor tolerance and immunotherapy will contribute to developing more effective inhibitors and/or drugs targeting Rac1.

### 3.2. Rac1 Regulates Cell Migration in Angiogenesis

Endothelial cell (EC) migration is important for angiogenesis. Rac1 and its downstream effector p21-activated kinases 1 (PAK1) play a role in EC migration [55]. During human umbilical vein EC (HUVEC) migration, Rac1 and other Rho GTPase can be activated by CD93, which is a key regulator of EC migration, promoting cell polarity and lamellipodia formation [56]. Acid α-glucosidase (GAA) deficiency causes autophagy disorder, which induces the upregulation of Rac1, thus resulting in aberrant tube formation and a migration increase in HUVEC [65].

Aberrant angiogenesis may induce vascular diseases such as retinal vascular diseases, which accompany the dysregulated migration of EC [66]. Age-related macular degeneration (AMD) is one of the retinal vascular diseases, and it is associated with choroidal EC (CEC) migrating to the retina to form macular neovascularization [67]. In the process, retinal pigment epithelium activates Rac1 in CEC by secreting vascular endothelial growth factor (VEGF), and then IQGAP1 binds to Rac1 to maintain its sustained activation, leading to CEC migration toward the retina [57]. Accumulation of 7-ketocholesterol is also an inducing factor of AMD which upregulates Rac1 activation in CEC [58]. Therefore, Rac1 is a potential target for developing antiangiogenic drugs. Studies show that sevoflurane is antiangiogenic; it can inhibit EC migration, adhesion and growth by suppressing Rac1 and RAS signaling to play its antiangiogenic role [58,59].

The above studies indicate that aberrant Rac1 activation and expression are key reasons for aberrant angiogenesis. Thus, Rac1 is a potential regulation target of angiogenesis in post-stroke recovery [55] and postmyocardial infarction cardiac repair [68], as well as the inhibition of angiogenesis in tumors [69].

### 3.3. Rac1 Regulates Cell Migration in Immune Response

In the process of immune response, immune cells migrate to the target site under the guidance of chemokines. Some immunomodulatory drugs, such as cereblon (CRBN), can regulate immune cell migration by activating Rac1 [52]. The migration speed and turning of T cells need to be restrained to avoid wasted movement. A previous study revealed that Arhgef6 can control Rac1 activity to repress actin polymerization and lamellipodia formation, which limits T-cell speed and turning [70]. Cells not only extend pseudopodia ahead during migration, but also retract cell uropods. Ramadass et al. found that Rac1-GTP trafficking in neutrophil uropods mediated by synaptotagmin-like 1 (JFC1) can promote uropod retraction, which helps with directional migration toward inflammation site [53]. Neutrophils in blood vessels transmigrate across the endothelium to enter inflamed tissue. Interestingly, they prefer asymmetric endothelial junctional membrane protrusions (JMPs) for transmigration, which are triggered by the local activation of Rac1, instead of randomly selecting exit sites [71].

Aberrant Rac1 activity is closely related to the development of autoimmune diseases. In the development of antineutrophil cytoplasmic antibody (ANCA)-associated vasculitis, neutrophils release extracellular vesicles (EVs) under the stimulation of ANCA, which downregulate Rac1 expression and migration in EC to interfere with vascular repair [72]. Increased aberrant Rac1 activity which causes an enhanced migratory phenotype in dendritic cells (DCs) is also in connection with allergic inflammation [73]. The inhibition of Rac1 activity can relieve inflammation. A study has shown that in oxazolone (OXZ)-induced Th2-type colitis, the cholinergic anti-inflammatory pathway inhibits Rac1 activity and eventual Rac1 inactivation of the colitis through the α7 nicotinic acetylcholine receptor (α7nAChR) on pDCs, which prevents pDCs migration toward the inflammation site [54]. These findings indicate that Rac1 is crucial for maintaining the normal function of immune cells. Rac1 is an important target for reducing inflammatory reaction and treating autoimmune diseases under pathological conditions. Furthermore, the inhibition of Rac1 activity can increase the recognition and cross presentation of DCs to tumor cells [74]. Thus, the inhibition of Rac1 activity is a prospective treatment in antitumor immunity. Proper immune response is helpful for successful biomaterial implantation [75]. Creating a favorable immune microenvironment for implants by regulating Rac1 activity needs to be further explored.

### 3.4. Rac1 Regulates Cell Migration in Other Physiological and Pathological Processes

Besides tumor invasion and metastasis, Rac1-regulated cell migration also plays an important role in other various physiological and pathological processes.

During body development, collective cell migration is a key link in various morphogenesis processes [76]. Defects in cell migration may cause some diseases such as brain diseases caused by neuronal migration disorders [77] and infertility caused by defects in germ cell migration [78]. Therefore, proper cell migration is the basis of proper body development. Rac1 activity level is highly relevant for proper cell migration. Dysregulated Rac1 activity in the nervous system can lead to serious neurodevelopmental disorders [79]. Neurodegenerative disorders such as Alzheimer’s disease and Parkinson’s disease are both related to aberrant Rac1-associated signal pathways [50,51]. Abnormally elevated Rac1 activity could alter the cell migration patterns to affect body development [80,81]. Thus, maintaining proper Rac1 activity is a key basis for normal development and homeostasis. A previous study has shown that Rac1 activity can be regulated by the ubiquitination pathway [82]. The inhibition of abnormal Rac1 activity through ubiquitination may be an important direction to follow to reduce the error rate of cell migration and alleviate the development disorders.

Cell migration is a key step in damage repair. Fibroblasts play an important role in damage repair. In the inflammatory phase of damage repair, fibroblasts activate and migrate to the wound site, during which multiple Rho GTPases such as Rac1 interact with fibers in ECM [83]. In addition to fibroblasts, skin wound healing also requires the collective migration of keratinocytes to the wound edge. The increased level of TGF-β1 at the wound increases miR-200b/c-3p inhibition, which accelerates the migration of keratinocytes to the wound site for repair [60]. Several complications of diabetes mellitus caused by chronic wound are life-threatening diseases. Mevastatin (a statin drug) can activate Rac1 through the epidermal growth factor (EGF) pathway that results in enhanced keratinocyte mobility and accelerated wound healing [61]. Dermal fibroblast migration impairment is a major cause of chronic wounds. Sirt1 (sirtuin 1) and Sirt3 (sirtuin 3) are potential drug targets, which can increase Rac1 expression to accelerate chronic wound healing [84]. Intestinal epithelial cell migration is necessary for intestinal wound healing. Amino acids in the intestine such as tryptophan can induce cell migration towards the wound site via the Rac1-related signaling pathway [62]. Rac1 also plays a role in fracture healing. The overexpression of family with sequence similarity 19 member A2 (TAFA2) at the site of a skeletal fracture recruits mesenchymal stem cells (MSCs) through the activation of Rac1/p38 signaling [63]. In addition, Rac1 plays a role in functional recovery after central nervous system (CNS) injury. The decrease in Rac1 expression hinders neural stem cell migration, inhibiting the functional recovery of the CNS [64]. Taken together, Rac1 is crucial for body damage repair. It is an important way to promote damage repair through regulating the activity and expression levels of Rac1, which can directly influence cell migration during damage repair. Furthermore, Rac1 involves the sensing of injury and the early activation of quiescent muscle stem cells [85], indicating Rac1 is closely related to stem-cell activation. Clarifying the molecular mechanisms is of great significance to damage repair and the treatment of many diseases.

## 4. Molecular Mechanisms of Cell Migration Regulated by Rac1

### 4.1. Rac1 Regulates Cell Front–Rear Polarity

Both single-cell migration and collective cell migration need the establishment of a front-to-rear polarity axis [86]. Jain et al. conducted collective cell migration experiments in an annular ring track designed by themselves, and employed PBD, a PAK binding protein, to be a fluorescent biosensor of active Rac1 [87]. The results showed that cells establish a unidirectional polarity gradient of Rac1 at single cell level. By interfering with the gradient, active Rac1 was thus distributed homogeneously within the whole cell, which led to random lamellipodia extensions and directional migration disorder. This indicated that the polarity gradient of Rac1 is essential for the establishment of cell polarity and subsequent directional migration.

The polarity distribution of Rac1 requires the regulation of Rac1 activation in space. Acentrosomal microtubules are involved in the delivery of Trio and other Rac1 GEFs to focal adhesion (FA), which results in excessive Rac1 activation around the cell periphery, leading to random lamellipodial protrusion and destruction of cell polarity. Centrosome can restrain the assembly of acentrosomal microtubules, thus repressing the excessive spatial activation of Rac1. Therefore, centrosome is helpful for maintaining Rac1 gradient and cell polarity [88]. T-lymphoma invasion and metastasis-1 (Tiam1) is the specific GEF of Rac1. One study has shown that Tiam1 performs asymmetrical accumulation at the leading edge of migrating cells with the existence of syndecan-4, which leads to the polarity distribution of Rac1-GTP and thus maintaining the directional persistence of cell migration. Knockout of syndecan-4 leads to a uniform distribution of Tiam1 and Rac1-GTP in cells, thereby inhibiting the formation of cell polarity and continuous directional cell migration [89].

The polarity gradient of Rac1 is not only regulated by the spatial distribution of GEFs, but also regulated by the spatial distribution of GAPs. As important sites of Rho signal transduction, FAs can isolate GEFs and GAPs at the cell periphery and center, respectively, forming the leading-edge Rac1 activity zone in migrating cells [90]. Interestingly, the peak value of Rac1 activation is not at the cell protruding edge, but at approximately 6 µm from the cell edge, then declines gradually from the site to the cell nucleus. β2-chimaerin has a direct role in shaping this distribution pattern of Rac1. It can be recruited by actin retrograde flow at the cell edge, where Rac1 activation is inhibited locally [32]. 

Taken together, these examples illustrate that the front-to-rear polarity axis of cells depends on building the polarity gradient of Rac1-GTP, which is crucial for initiating cell migration and modulated by a variety of active regulatory factors spatially. It would be of great significance to explore the regulatory mechanisms in depth and lay a theoretical foundation for the subtle regulation of Rac1 activity.

### 4.2. Rac1-Scar/Wave-Arp2/3 Signaling Pathway

Both cell polarity and cell migration require pseudopodium formation [91]. The Scar/Wave complex, consisting of five subunits, is a key factor to promote the formation of actin-rich protrusions in migrating cells. As an actin-related protein 2/3 (Arp2/3) activator, it stimulates actin polymerization through the Arp2/3 complex to generate lamellipodia and pseudopodia [92]. 

The NCKAP1 subunit of the Scar/Wave complex is a major downstream target of Rac1. Rac1-Scar/Wave-Arp2/3 is of great importance for tumor cell movement. One study shows that the targeting of NCKAP1 to block Rac1-Scar/Wave signaling can slow tumor progression and possibly enhance immune infiltration [36]. Upregulation of active Rac1 and Arp2/3 can induce F-actin cytoskeletal remodeling in HCC cells, promoting cell migration, invasion, and metastasis [93]. Thus, targeting Rac1-Scar/Wave-Arp2/3 would help to inhibit tumor progression. Another study shows that Rac1-Scar/Wave-Arp2/3 can be negatively regulated by Nance-Horan Syndrome-like 1 protein (NHSL1), which directly interacts with Scar/Wave, thus reducing cell lamellipodia stability and migration efficiency [94]. As a local inhibitor of lamellipodia induced by Scar/Wave, CYFIP-related Rac interactor (CYRI) can compete with Scar/Wave to bind Rac1, which is important in regulating pseudopod complexity and dynamics [95]. Targeting negative regulatory factors can indirectly regulate Rac1-Scar/Wave-Arp2/3 to suppress tumor metastasis.

In addition, Rac1-Scar/Wave-Arp2/3 also play a role in physiological cell migration. Some drugs, such as dragon’s blood (DB), can exogenously upregulate Rac1-Scar/Wave-Arp2/3 pathway activity, thus facilitating F-actin polymerization, which is crucial for the formation of focal adhesions and intestinal epithelial cell migration [96]. Moreover, Rac1 is involved in milk fat globule-epidermal growth factor 8 (MFG-E8)-mediated Arp2 expression, which mediates actin polymerization during VSMC migration [97].

Altogether, after receiving upstream signals from Rac1, the Scar/Wave complex regulates F-actin cytoskeleton polymerization by activating Arp2/3 complex, thus forming actin-based membrane processes, which are essential for cell migration and cancer cell invasion. Rac1-Scar/Wave-Arp2/3 is a key target for preventing or inhibiting cancer invasion and metastasis.

### 4.3. Rac1/PAK1 Signaling Pathway

PAK1 is a member of the serine-threonine kinase family. PAK1 was considered an interactor of Rac1 initially [98]. With the deepening of research, PAK1 is gradually becoming recognized as an important effector downstream of Rac1 [14]. The Rac1/PAK1 signaling pathway is the most established mechanism for RAC1-mediated cytoskeleton reorganization, playing an important role in cell movement [6].

Rac1/PAK1 primarily functions in tumor metastasis. Both the GEFT [99] and DEP domains containing 1B (DEPDC1B) [100] can interact with Rac1 to activate Rac1 and downstream PAK1, thus promoting EMT and enhancing the invasion and migration of tumor cells. LIMK1/cofilin, downstream of Rac1/PAK1, is one of the major ways for Rho GTPase to regulate actin cytoskeleton. One study has demonstrated that DEPDC1B can promote tumor cell invasion and migration by activating the Rac1/PAK1/LIMK1/cofilin pathway [101].

Because of its crucial role in tumor metastasis, Rac1/PAK1 is widely used as a target for antitumor treatment. Some miRNAs, such as miR-142-3p, inhibit tumor cell migration by restraining Rac1/PAK1 [102]. Chlorine e6 photodynamic therapy (Ce6-PDT) is a new method of tumor treatment. Recent studies have shown that Ce6-PDT inhibits tumor cell migration by destroying the structure of actin cytoskeleton and reducing lamellipodium formation, which is closely related to the downregulation of Rac1/PAK1/LIMK1/cofilin [103]. The inhibition of Rho GDP differentiation inhibitor 2 (RhoGDI2) can also reduce tumor cell migration by downregulating Rac1/PAK1/LIMK1 [104]. In addition to LIMK/cofilin, β-catenin also plays a role in cell migration as a downstream effector of Rac1/PAK1. Centchroman (CC), proven to have antitumor activity, prevents β-catenin from entering the cell nucleus to regulate the transcription of migration-related genes by suppressing Rac1/PAK1/β-catenin. As a member of the GEF family, Vav2 can also regulate the activation of Rac1/PAK1 [105]. It was found that miR-331-3p inhibits Rac1/PAK1/β-catenin by targeting Vav2, thereby inhibiting EMT and the subsequent invasion and migration of tumor cells [106].

**Figure 2:**
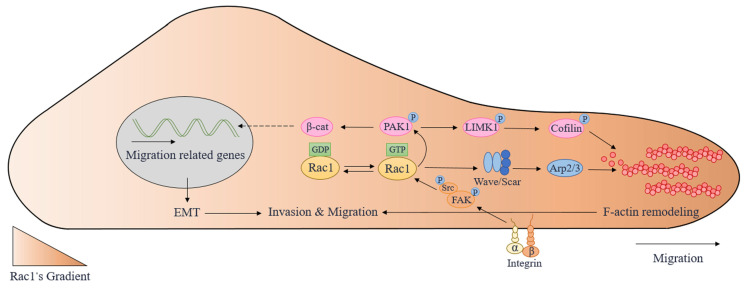
The molecular mechanisms involved in cell migration regulated by Rac1. Arp2/3: actin-related protein 2/3; EMT: epithelial–mesenchymal transition; FAK: focal adhesion kinase; LIMK1: LIM domain kinase 1; PAK1: p21-activated kinases 1; Rac1: Ras-related C3 botulinum toxin substrate 1; β-cat: β-catenin.

Taken together, LIMK1/cofilin is a crucial downstream signaling pathway of Rac1/PAK1 for regulating the rearrangement of actin cytoskeleton and lamellipodia extension of tumor cells. Through β-catenin, Rac1/PAK1 can facilitate EMT (Figure 2), which is an essential condition for the invasion and metastasis of tumor cells. Thus, Rac1/PAK1 can be an important target for antitumor treatment.

### 4.4. Src/FAK/Rac1 Signaling Pathway

FAK is a non-receptor tyrosine kinase located at FAs, which can receive signals from ECM through integrins and then activate downstream signal transduction [107]. FAK often forms a complex with Src to activate downstream effectors such as Rac1. The activation of Src/FAK plays an important role in tumor cell metastasis and normal cell movement [108].

In hepatitis B virus (HBV)-associated hepatocellular carcinoma, the accumulation of ROS induced by HBV enhances the combination of IQGAP1 and Rac1, which activate the Rac1 and Src/FAK pathway to enhance anoikis resistance and the migration of hepatoma cells [109]. The transmembrane protein inositol-requiring enzyme 1α (IRE1α) in endoplasmic reticulum has been proved to be involved in tumor metastasis. Its protein product X-box binding protein 1 (XBP1) regulates the expression of fibronectin-1 (FN1) at the mRNA level, which transmits signals to Src/FAK/Rac1 via integrins [110]. By downregulating the integrin-triggered FAK/Src signaling pathway, integrin can inhibit EMT and attenuate the invasion and migration of breast cancer cells [111]. The signals transmitted from integrins to FAK can also activate Rac1 through PI3K/Akt, inducing actin cytoskeleton rearrangement and accelerating cell migration [112].

During body development, Wnt5b regulates the activation of Src/FAK/Rac1 through integrins, thus mediating actin polymerization and subsequent cell migration [113]. In microglia, under the stimulation of neural injury or amyloid-β (Aβ), Src/FAK/Rac1 is activated, which is capable of promoting actin cytoskeleton rearrangement and microglial migration towards injury for repair or for Aβ plaque removal [50].

To sum up, both physiological and pathological cell migration is related to the Src/FAK/Rac1 pathway. After receiving extracellular migration signals, integrins or other cell membrane receptors integrate and transmit them to Src/FAK/Rac1, facilitating cell migration through downstream actin cytoskeleton rearrangement (Figure 2).

## 5. Inhibition of Rac1 Activity in Cancer Therapy

The level of Rac1 activity is important for its function in tumor cell migration, invasion and metastasis. Therefore, restraining Rac1 activation is the key to cancer therapy.

Multiple selective inhibitors of Rac1 have been developed, such as NSC23766 and EHT 1864, that were designed to target different sites of Rac1 [7,114,115]. Some more potent and clinically effective inhibitors, such as MBQ-167, are in preclinical development for the treatment of advanced solid tumors, but there is still a long way to go before they can be truly applied in clinical cancer therapy [7]. The aberrant activity of Rac1 and its active regulatory factors are widely found in multiple cancers. Since GEF-induced activation is the most common mechanism for Rac1 activation, the development of Rac1 GEF inhibitors is a promising strategy in cancer therapy. Ciarlantini et al. found that compound 1D-142 can inhibit Tiam1-induced Rac1 activation, thus decreasing proliferation, migration and inducing the apoptotic cell death program of non-small cell lung cancer (NSCLC) cells [116]. Inosine monophosphate dehydrogenase 2 (IMPDH2) is fundamental for regulating GTP levels in live cells, which closely correlate with Rac1 activity. The substitution of endogenous IMPDH2 with IMPDH2 mutants incapable of binding Rac1 can suppress Rac1 activity and decrease cell invasion [117].

## 6. Conclusions and Perspectives

Cell migration is crucial in physiology, development and disease-related processes, for which it is an essential basis for modulating these biological processes. Errors in cell migration may cause devastating consequences, such as defects in body development, nerve dysfunction, chronic wounds, immune deficiency and tumor metastasis. As a key player in regulating cell migration, the disorder of Rac1 activity is one of the main factors in aberrant cell migration. Owing to its high expression in multiple invasive tumors, Rac1 is identified as a key target for antitumor therapy. In recent years, researchers have carried out many investigations on the important role of Rac1 in cell migration, and have made significant research progress. With an in-depth understanding of Rac1′s contribution to cell migration, intervention in various physiological and pathological processes related to cell migration and tumor metastasis by regulating the activity of Rac1 is to be expected.

At present, the important roles of Rac1 in regulating cell migration and tumor metastasis under physiological and pathological conditions have been recognized. However, the molecular mechanisms of Rac1 regulating cell migration are very complex and many details are still elusive, which make the understanding of Rac1′s role in cell migration still fragmented and one-sided. There are still many problems in this area to be further solved. In the future, the following problems need to be explored: (1) how the activity regulators regulate Rac1 activation spatially and temporally, and maintain the dynamic balance of active Rac1 in specific subcellular regions; (2) how Rac1 interacts with RhoA, Cdc42 and other Rho GTPases spatially and temporally to realize the dynamic regulation of the cytoskeleton; (3) how Rac1 interacts with various upstream and downstream signal molecules; (4) the development of antitumor therapeutic drugs targeting Rac1 (Rac1 activity inhibitors) has been reported; however, the clinical applications show non-negligible limitations and side effects. Therefore, it still needs further research and a breakthrough. It is believed that with the deepening of the study into the mechanisms of how Rac1 regulates cell migration, a variety of factors that influence Rac1 activity and the integrated signal-regulation network of cell migration will be clarified. It should be possible to precisely regulate Rac1 activity during cell migration, which would be of great significance for the prevention and treatment of cancers and other relevant diseases.

## Figures and Tables

**Table 1:** Cell migration regulated by Rac1 and the involved direct or indirect signaling pathways.

Cell Types		Cell Lines	Signaling Pathways	Ref.
Tumor cells	HNSC cells	UM-SCC-1, UM-SCC-17B	Rap1/α5β1-integrin/Rac1	[34]
	HCC cells	MHCC-97H, MHCC-LM9, VETC-2	AR/Rac1	[35]
		SNU-387, SNU-423, SNU-449, SNU-475, Hep3B, HepG2	Akt/Rac1/Fascin-1/Hippo/YAP	[45]
	CM cells	\	Rac1/Scar/Wave	[36]
	OS cells	MG-63	Ras/Rac1/Wave2	[37]
	RMS cells	SMS-CTR, RD, Rh5, Rh30	HDAC6/Rac1	[38]
	PAAD cells	MIA, PaCa-2, PANC-1	Myosin II/Rac1	[39]
	Cervical cancer cells	Hela, SiHa, Caski	DTL/Rac1/JNK/FOXO1	[41]
	COAD/READ cells	SW480, HCT116	TGF-β1/aPKC-ι/Rac1/JNK	[42]
		HCT116	Pals1/Arf6/Rac1	[49]
	Gastric cancer cells	BGC-823, SGC-7901	NEDD9/MICAL1/Rac1	[43]
	Breast cancer cells	MDA-MB-231, MDA-MB-46	SOX2/NEDD9/Rac1/HIF-1α	[44]
	GBM cells	M059K, M059J, U87	DNA-PKcs/Akt2/Rac1	[46]
	Bladder cancer cells	RT4, 5637, T24	KDM6A/ARHGDIB/Rac1	[48]
Immune cells	Microglia	\	TREM2/FAK/Rac1	[50]
		BV-2	CXCL12/CXCR4/FAK/Src/Rac1	[51]
	NK cells	NK-92	CRBN/Rac1	[52]
	Neutrophils	\	JFC1/Rac1	[53]
	DC cells	pDC	α7nAChR/JAK2/STAT3/casepase-3/Rac1	[54]
Other cells	ECs	HEBC-5i	Rac1/PAK1	[55]
		\	CD93/Cbl/Crk/Rac1	[56]
		CEC	IQGAP1/Rac1	[57]
			7KC/Rac1	[58]
		HUVEC	Rac1/paxillin/FAK	[59]
	Keratinocytes	HaCaT2	TGF-β1/miR-200b/c-3p/Rac1	[60]
		\	EGF/EGFR/Rac1	[61]
	Epithelial cells	IPEC-J2	Trp/CaSR/Rac1/PLC-γ1	[62]
	MSCs	hMSC	TAFA2/Rac1/p38	[63]
	NSCs	NSC	α-SMA/Rac1/F-actin	[64]

AR: androgen receptor; Akt: serine/threonine kinase; aPKC-ι: atypical protein kinase C-ι; Akt2: Akt serine/threonine kinase 2; ARHGDIB: Rho GDP dissociation inhibitor β; CM: cutaneous melanoma; COAD/READ: colon adenocarcinoma/rectum adenocarcinoma esophageal carcinoma; CXCL12: CXC chemokine ligand 12; CXCR4: CXC chemokine receptor type 4; CRBN: cereblon; Syk: spleen tyrosine kinase; CaSR: calcium-sensing receptor; Cbl: Casitas B cell lymphoma protein; DC: dendritic; DTL: denticleless E3 ubiquitin protein ligase homolog; DNA-PKcs: DNA-dependent protein kinase catalytic subunit; eIF2α-P: phosphorylation of the α subunit of eukaryotic initiation factor 2; EC: endothelial cell; EGF: epidermal growth factor; EGFR: epidermal growth factor receptor; FOXO1: forkhead box protein O1; FAK: focal adhesion kinase; GBM: glioblastoma; HNSC: head and neck squamous cell carcinoma; HCC: hepatocellular carcinoma; HDAC6: histone deacetylase 6; HIF1α: hypoxia inducible factor-1α; IQGAP1: IQ protein motif containing GTPase activating protein 1; JNK: c-Jun N-terminal kinase; JFC1: synaptotagmin-like 1; JAK2: janus kinase 2; KDM6A: lysine demethylase 6A; MSC: mesenchymal stem cell; MICAL1: molecules interacting with Cas L1; NK: natural killer; NSC: neural stem cell; NEDD9: neural precursor cell expressed, developmentally downregulated protein 9; OS: osteosarcoma; PAAD: pancreatic adenocarcinoma; PLC-γ1: phospholipase C-γ1; PAK1: p21-activated kinases 1; RMS: rhabdomyosarcoma; Rap1: Ras-associated protein 1; Rac1: Ras-related C3 botulinum toxin substrate 1; RhoGDI2: Rho GDP dissociation inhibitor 2; ROS: reactive oxygen species; STAT3: signal transducer and activator of transcription 3; TGF-β1: transforming growth factor-β1; TREM2: triggering receptor expressed on myeloid cells 2; Trp: tryptophan; TAFA2: family with sequence similarity 19 member A2; Wave: WASP family verprolin homologous protein; Wave2: WASP family verprolin homologous protein 2; YAP: Yes-associated protein; α7nAChR: α7 nicotinic acetylcholine receptor; α-SMA: α smooth muscle actin; 7KC: 7-ketocholesterol.

## Data Availability

All data are contained within the article.
